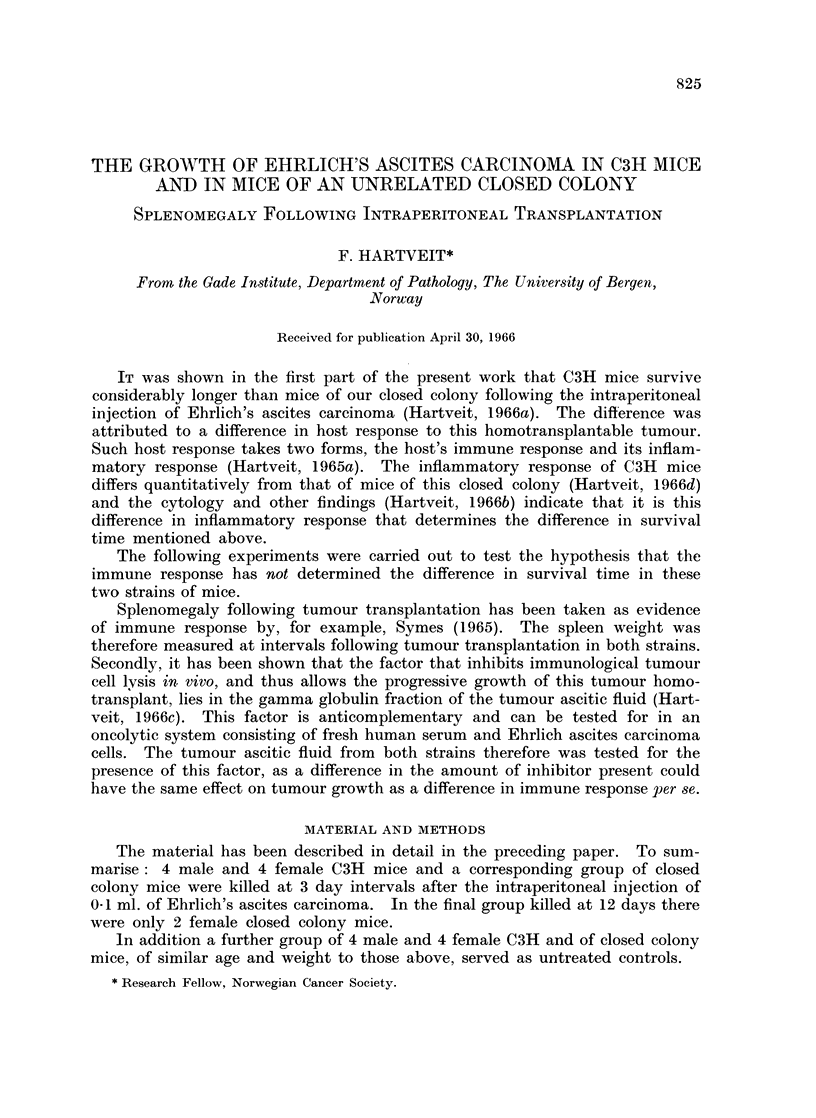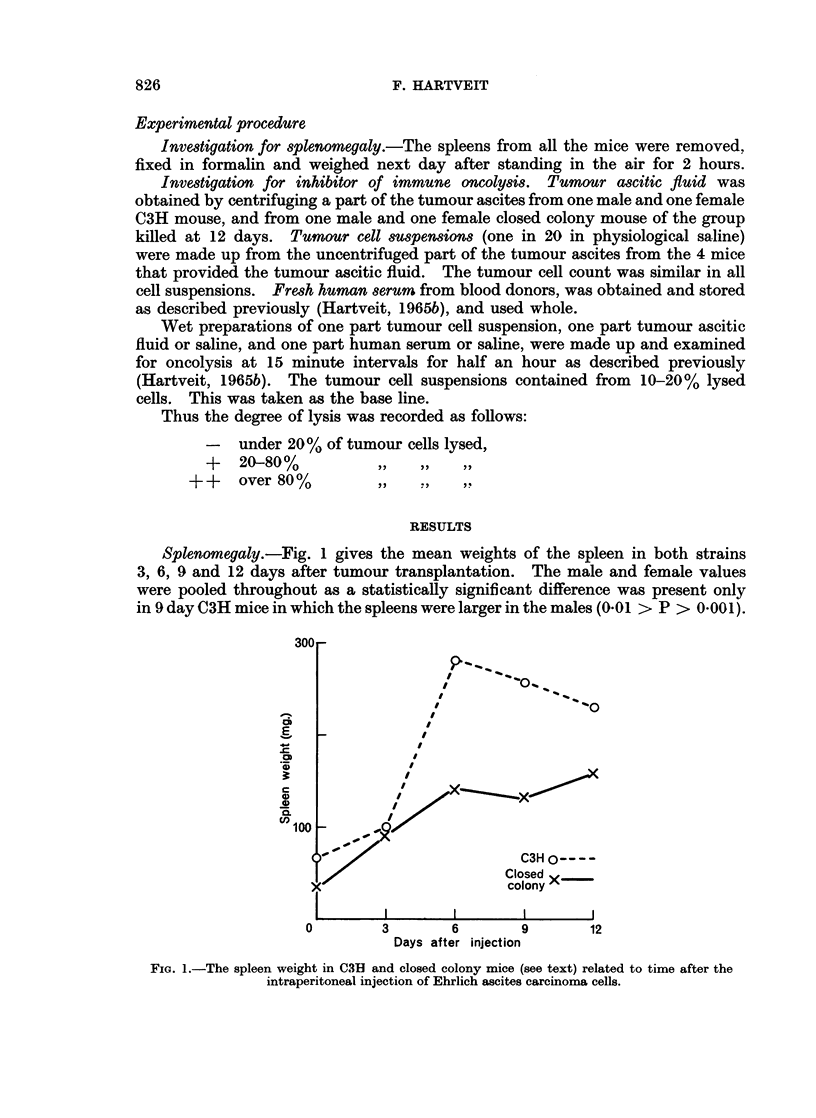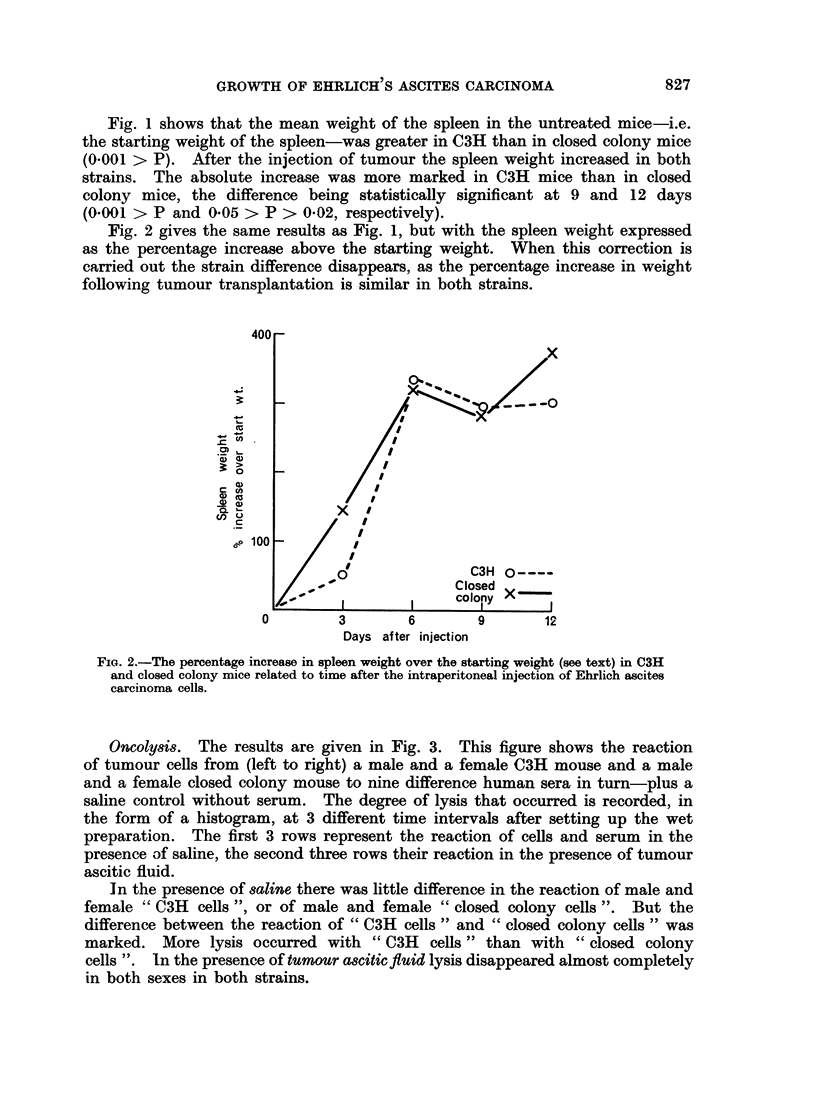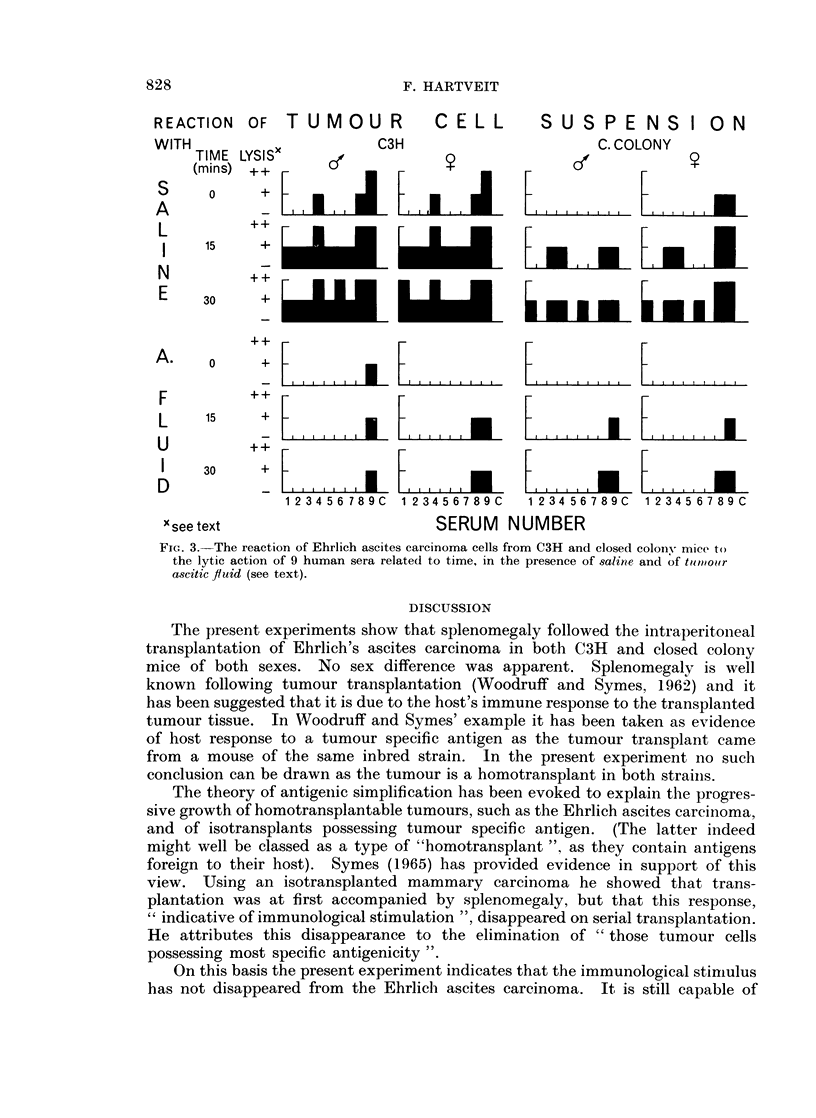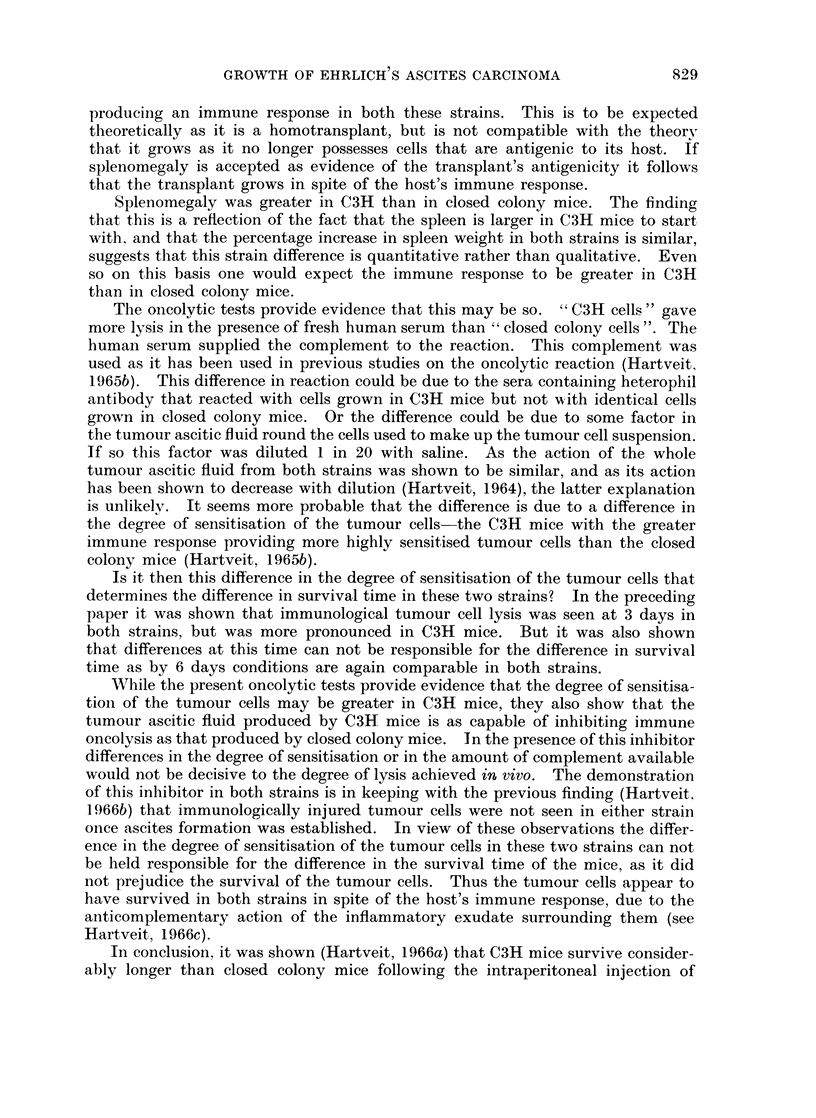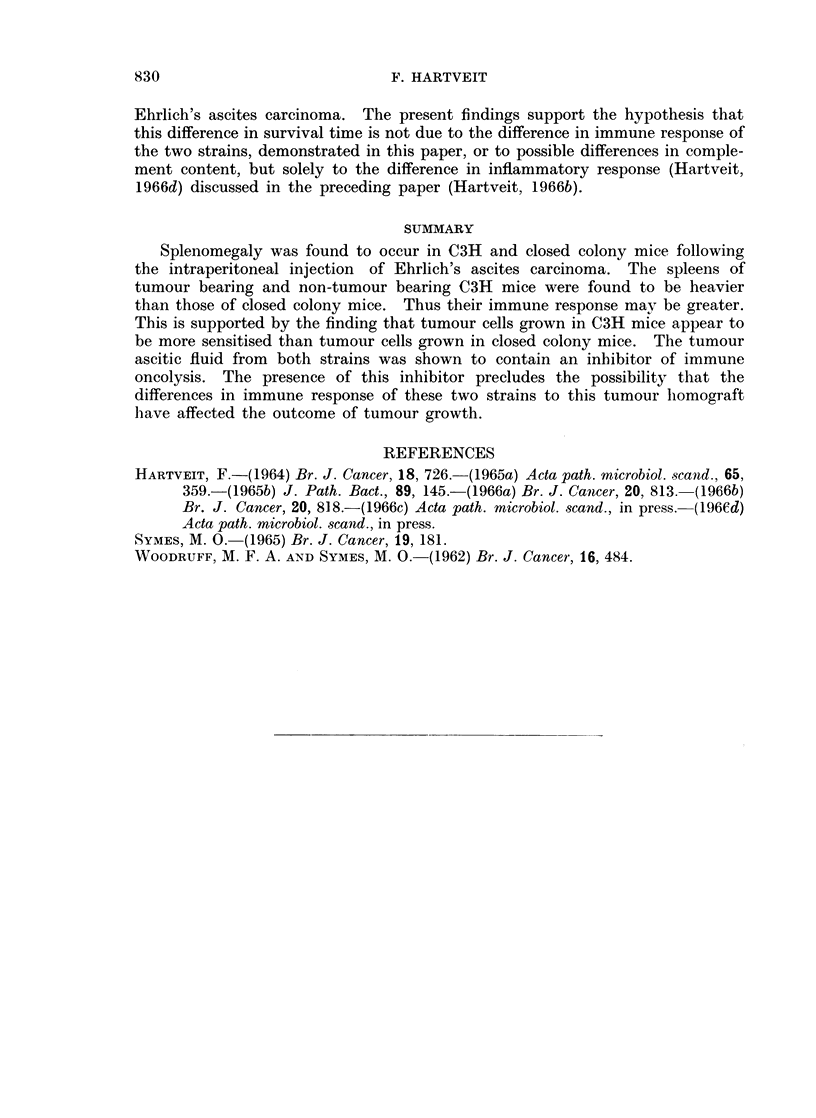# The growth of Ehrlich's ascites carcinoma in C3H mice and in mice of an unrelated closed colony. Splenomegaly following intraperitoneal transplantation.

**DOI:** 10.1038/bjc.1966.94

**Published:** 1966-12

**Authors:** F. Hartveit


### THE GROWTH OF EHRLICH'S ASCITES CARCINOMA IN C3H MICE AND IN MICE OF AN UNRELATED CLOSED COLONY

### SPLENOMEGALY FOLLOWING INTRAPERITONEAL TRANSPLANTATION

F. HARTVEIT*

*From the Gade Institute, Department of Pathology, The University of Bergen, Norway*

Received for publication April 30, 1966

IT was shown in the first part of the present work that C3H mice survive considerably longer than mice of our closed colony following the intraperitoneal injection of Ehrlich's ascites carcinoma (Hartveit, 1966*a*). The difference was attributed to a difference in host response to this homotransplantable tumour. Such host response takes two forms, the host's immune response and its inflammatory response (Hartveit, 1965*a*). The inflammatory response of C3H mice differs quantitatively from that of mice of this closed colony (Hartveit, 1966*d*) and the cytology and other findings (Hartveit, 1966*b*) indicate that it is this difference in inflammatory response that determines the difference in survival time mentioned above.

The following experiments were carried out to test the hypothesis that the immune response has *not* determined the difference in survival time in these two strains of mice.

Splenomegaly following tumour transplantation has been taken as evidence of immune response by, for example, Symes (1965). The spleen weight was therefore measured at intervals following tumour transplantation in both strains. Secondly, it has been shown that the factor that inhibits immunological tumour cell lysis *in vivo*, and thus allows the progressive growth of this tumour homotransplant, lies in the gamma globulin fraction of the tumour ascitic fluid (Hartveit, 1966*c*). This factor is anticomplementary and can be tested for in an oncolytic system consisting of fresh human serum and Ehrlich ascites carcinoma cells. The tumour ascitic fluid from both strains therefore was tested for the presence of this factor, as a difference in the amount of inhibitor present could have the same effect on tumour growth as a difference in immune response *per se*.

### MATERIAL AND METHODS

The material has been described in detail in the preceding paper. To summarise: 4 male and 4 female C3H mice and a corresponding group of closed colony mice were killed at 3 day intervals after the intraperitoneal injection of 0·1 ml. of Ehrlich's ascites carcinoma. In the final group killed at 12 days there were only 2 female closed colony mice.

In addition a further group of 4 male and 4 female C3H and of closed colony mice, of similar age and weight to those above, served as untreated controls.

* Research Fellow, Norwegian Cancer Society.

*Experimental procedure*

*Investigation for splenomegaly.*—The spleens from all the mice were removed, fixed in formalin and weighed next day after standing in the air for 2 hours.

*Investigation for inhibitor of immune oncolysis. Tumour ascitic fluid* was obtained by centrifuging a part of the tumour ascites from one male and one female C3H mouse, and from one male and one female closed colony mouse of the group killed at 12 days. *Tumour cell suspensions* (one in 20 in physiological saline) were made up from the uncentrifuged part of the tumour ascites from the 4 mice that provided the tumour ascitic fluid. The tumour cell count was similar in all cell suspensions. *Fresh human serum* from blood donors, was obtained and stored as described previously (Hartveit, 1965*b*), and used whole.

Wet preparations of one part tumour cell suspension, one part tumour ascitic fluid or saline, and one part human serum or saline, were made up and examined for oncolysis at 15 minute intervals for half an hour as described previously (Hartveit, 1965*b*). The tumour cell suspensions contained from 10–20% lysed cells. This was taken as the base line.

Thus the degree of lysis was recorded as follows:

| | |
|---|---|
| — | under 20% of tumour cells lysed, |
| + | 20–80% ,, ,, ,, |
| ++ | over 80% ,, ,, ,, |

### RESULTS

*Splenomegaly.*—Fig. 1 gives the mean weights of the spleen in both strains 3, 6, 9 and 12 days after tumour transplantation. The male and female values were pooled throughout as a statistically significant difference was present only in 9 day C3H mice in which the spleens were larger in the males ($0{\cdot}01 > P > 0{\cdot}001$).

FIG. 1.—The spleen weight in C3H and closed colony mice (see text) related to time after the intraperitoneal injection of Ehrlich ascites carcinoma cells.

Fig. 1 shows that the mean weight of the spleen in the untreated mice—i.e. the starting weight of the spleen—was greater in C3H than in closed colony mice ($0{\cdot}001 > P$). After the injection of tumour the spleen weight increased in both strains. The absolute increase was more marked in C3H mice than in closed colony mice, the difference being statistically significant at 9 and 12 days ($0{\cdot}001 > P$ and $0{\cdot}05 > P > 0{\cdot}02$, respectively).

Fig. 2 gives the same results as Fig. 1, but with the spleen weight expressed as the percentage increase above the starting weight. When this correction is carried out the strain difference disappears, as the percentage increase in weight following tumour transplantation is similar in both strains.

FIG. 2.—The percentage increase in spleen weight over the starting weight (see text) in C3H and closed colony mice related to time after the intraperitoneal injection of Ehrlich ascites carcinoma cells.

*Oncolysis.* The results are given in Fig. 3. This figure shows the reaction of tumour cells from (left to right) a male and a female C3H mouse and a male and a female closed colony mouse to nine difference human sera in turn—plus a saline control without serum. The degree of lysis that occurred is recorded, in the form of a histogram, at 3 different time intervals after setting up the wet preparation. The first 3 rows represent the reaction of cells and serum in the presence of saline, the second three rows their reaction in the presence of tumour ascitic fluid.

In the presence of *saline* there was little difference in the reaction of male and female " C3H cells ", or of male and female " closed colony cells ". But the difference between the reaction of " C3H cells " and " closed colony cells " was marked. More lysis occurred with " C3H cells " than with " closed colony cells ". In the presence of *tumour ascitic fluid* lysis disappeared almost completely in both sexes in both strains.

FIG. 3.—The reaction of Ehrlich ascites carcinoma cells from C3H and closed colony mice to the lytic action of 9 human sera related to time, in the presence of *saline* and of *tumour ascitic fluid* (see text).

### DISCUSSION

The present experiments show that splenomegaly followed the intraperitoneal transplantation of Ehrlich's ascites carcinoma in both C3H and closed colony mice of both sexes. No sex difference was apparent. Splenomegaly is well known following tumour transplantation (Woodruff and Symes, 1962) and it has been suggested that it is due to the host's immune response to the transplanted tumour tissue. In Woodruff and Symes' example it has been taken as evidence of host response to a tumour specific antigen as the tumour transplant came from a mouse of the same inbred strain. In the present experiment no such conclusion can be drawn as the tumour is a homotransplant in both strains.

The theory of antigenic simplification has been evoked to explain the progressive growth of homotransplantable tumours, such as the Ehrlich ascites carcinoma, and of isotransplants possessing tumour specific antigen. (The latter indeed might well be classed as a type of "homotransplant ", as they contain antigens foreign to their host). Symes (1965) has provided evidence in support of this view. Using an isotransplanted mammary carcinoma he showed that transplantation was at first accompanied by splenomegaly, but that this response, " indicative of immunological stimulation ", disappeared on serial transplantation. He attributes this disappearance to the elimination of " those tumour cells possessing most specific antigenicity ".

On this basis the present experiment indicates that the immunological stimulus has not disappeared from the Ehrlich ascites carcinoma. It is still capable of producing an immune response in both these strains. This is to be expected theoretically as it is a homotransplant, but is not compatible with the theory that it grows as it no longer possesses cells that are antigenic to its host. If splenomegaly is accepted as evidence of the transplant's antigenicity it follows that the transplant grows in spite of the host's immune response.

Splenomegaly was greater in C3H than in closed colony mice. The finding that this is a reflection of the fact that the spleen is larger in C3H mice to start with, and that the percentage increase in spleen weight in both strains is similar, suggests that this strain difference is quantitative rather than qualitative. Even so on this basis one would expect the immune response to be greater in C3H than in closed colony mice.

The oncolytic tests provide evidence that this may be so. "C3H cells" gave more lysis in the presence of fresh human serum than "closed colony cells". The human serum supplied the complement to the reaction. This complement was used as it has been used in previous studies on the oncolytic reaction (Hartveit, 1965*b*). This difference in reaction could be due to the sera containing heterophil antibody that reacted with cells grown in C3H mice but not with identical cells grown in closed colony mice. Or the difference could be due to some factor in the tumour ascitic fluid round the cells used to make up the tumour cell suspension. If so this factor was diluted 1 in 20 with saline. As the action of the whole tumour ascitic fluid from both strains was shown to be similar, and as its action has been shown to decrease with dilution (Hartveit, 1964), the latter explanation is unlikely. It seems more probable that the difference is due to a difference in the degree of sensitisation of the tumour cells—the C3H mice with the greater immune response providing more highly sensitised tumour cells than the closed colony mice (Hartveit, 1965*b*).

Is it then this difference in the degree of sensitisation of the tumour cells that determines the difference in survival time in these two strains? In the preceding paper it was shown that immunological tumour cell lysis was seen at 3 days in both strains, but was more pronounced in C3H mice. But it was also shown that differences at this time can not be responsible for the difference in survival time as by 6 days conditions are again comparable in both strains.

While the present oncolytic tests provide evidence that the degree of sensitisation of the tumour cells may be greater in C3H mice, they also show that the tumour ascitic fluid produced by C3H mice is as capable of inhibiting immune oncolysis as that produced by closed colony mice. In the presence of this inhibitor differences in the degree of sensitisation or in the amount of complement available would not be decisive to the degree of lysis achieved *in vivo*. The demonstration of this inhibitor in both strains is in keeping with the previous finding (Hartveit, 1966*b*) that immunologically injured tumour cells were not seen in either strain once ascites formation was established. In view of these observations the difference in the degree of sensitisation of the tumour cells in these two strains can not be held responsible for the difference in the survival time of the mice, as it did not prejudice the survival of the tumour cells. Thus the tumour cells appear to have survived in both strains in spite of the host's immune response, due to the anticomplementary action of the inflammatory exudate surrounding them (see Hartveit, 1966*c*).

In conclusion, it was shown (Hartveit, 1966*a*) that C3H mice survive considerably longer than closed colony mice following the intraperitoneal injection of

Ehrlich's ascites carcinoma. The present findings support the hypothesis that this difference in survival time is not due to the difference in immune response of the two strains, demonstrated in this paper, or to possible differences in complement content, but solely to the difference in inflammatory response (Hartveit, 1966*d*) discussed in the preceding paper (Hartveit, 1966*b*).

### SUMMARY

Splenomegaly was found to occur in C3H and closed colony mice following the intraperitoneal injection of Ehrlich's ascites carcinoma. The spleens of tumour bearing and non-tumour bearing C3H mice were found to be heavier than those of closed colony mice. Thus their immune response may be greater. This is supported by the finding that tumour cells grown in C3H mice appear to be more sensitised than tumour cells grown in closed colony mice. The tumour ascitic fluid from both strains was shown to contain an inhibitor of immune oncolysis. The presence of this inhibitor precludes the possibility that the differences in immune response of these two strains to this tumour homograft have affected the outcome of tumour growth.

### REFERENCES

Hartveit, F.—(1964) *Br. J. Cancer*, **18**, 726.—(1965*a*) *Acta path. microbiol. scand.*, **65**, 359.—(1965*b*) *J. Path. Bact.*, **89**, 145.—(1966*a*) *Br. J. Cancer*, **20**, 813.—(1966*b*) *Br. J. Cancer*, **20**, 818.—(1966*c*) *Acta path. microbiol. scand.*, in press.—(1966*d*) *Acta path. microbiol. scand.*, in press.

Symes, M. O.—(1965) *Br. J. Cancer*, **19**, 181.

Woodruff, M. F. A. and Symes, M. O.—(1962) *Br. J. Cancer*, **16**, 484.